# Estimating the size of urban populations using Landsat images: a case study of Bo, Sierra Leone, West Africa

**DOI:** 10.1186/s12942-019-0180-1

**Published:** 2019-07-11

**Authors:** Roger Hillson, Austin Coates, Joel D. Alejandre, Kathryn H. Jacobsen, Rashid Ansumana, Alfred S. Bockarie, Umaru Bangura, Joseph M. Lamin, David A. Stenger

**Affiliations:** 1 Washington, DC USA; 2 Ground Up Spectral Solutions, Inc., Salt Lake City, UT USA; 3 Information Technology Division, Naval Research Laboratory, Washington, DC USA; 4 Department of Global and Community Health, George Mason University, Fairfax, VA USA; 5 Njala University, Bo, Sierra Leone; 6 Mercy Hospital Research Laboratory, Bo, Sierra Leone; 7 Center for Bio/Molecular Science and Engineering, Naval Research Laboratory, Washington, DC USA

**Keywords:** Remote Sensing, Population Estimation, Spatial Epidemiology, Landsat, Sierra Leone, MCMC, urban footprint

## Abstract

**Background:**

This is the third paper in a 3-paper series evaluating alternative models for rapidly estimating neighborhood populations using limited survey data, augmented with aerial imagery.

**Methods:**

Bayesian methods were used to sample the large solution space of candidate regression models for estimating population density.

**Results:**

We accurately estimated the population densities and counts of 20 neighborhoods in the city of Bo, Sierra Leone, using statistical measures derived from Landsat multi-band satellite imagery. The best regression model proposed estimated the latter with an absolute median proportional error of 8.0%, while the total population of the 20 neighborhoods was estimated with an error of less than 1.0%. We also compare our results with those obtained using an empirical Bayes approach.

**Conclusions:**

Our approach provides a rapid and effective method for constructing predictive models for population densities and counts utilizing remote sensing imagery. Our results, including cross-validation analysis, suggest that masking non-urban areas in the Landsat section images prior to computing the candidate covariate regressors should further improve model generality.

## Introduction

In resource-limited environments, it is desirable to be able to rapidly estimate the density of local populations. The ability to estimate population sizes is important in places where population growth is relatively high and census data are relatively old. Many of these locations are in urbanizing areas of low- and lower-middle-income countries.

Such estimates are invaluable for health planning, refugee support [1], epidemiological modeling [2], and for state and municipality-sponsored allocation of public resources and services. Most commonly, such estimates are made using some combination of aerial imagery and local survey data. In two recent papers, we used ground-truth survey data from Bo, Sierra Leone, to model several different approaches for estimating section (neighborhood) population. As a function of sample size, comparisons were made between the uncertainty of the estimated population based on the mean occupancy of residential structures and the mean number of individuals per square meter of rooftop area [3, 4].

Both studies required only a limited amount of survey data, in addition to estimates of the total number of residential structures in a region of interest. Methods that utilize rooftop area additionally require estimates of individual and total rooftop areas in regions of interest. In our current study, we examine the possibility of using Landsat 5 *thematic mapper* (TM) data to estimate the population densities of sections in Bo, Sierra Leone, without the necessity of either explicitly estimating the number of individual residential structures present nor a requirement to extract and estimate rooftop areas.

### Description of the study area

Bo is Sierra Leone’s second largest city, and its population and footprint has grown substantially over the past two decades. The city of Bo itself is approximately 30.10 km^2^ in area, and is divided into 68 mutually-exclusive neighborhoods or *sections* [2]. These sections vary in size from 0.02 to 2.33 km^2^. For 20 of the 68 sections, residential survey data collected in 2011 are available [3] as summarized in Table 1.

**Table 1:** Bo municipal survey data

(1) Section	(2) Persons	(3) Area (km$^2$)	(4) Persons/area	(5) Residential structures	(6) Total structures
Moibawo Farm	135	0.5	270.04	17	43
Roma	139	0.04	3510.72	4	52
Bo Central	273	0.066	4137.9	33	103
Toubu	454	0.016	28089.66	34	46
Salina	580	0.467	1242.8	59	231
Dodo	597	0.049	12126.22	26	88
Reservation	637	2.329	273.5	66	252
Kpetewoma	640	0.197	3250.74	46	105
Lewabu	879	0.479	1836.16	105	117
Tengbewabu	1068	0.68	1571.17	136	233
New York	1088	1.513	719.3	116	605
Komende	1103	0.196	5622.16	56	258
Kindia Town	1160	0.146	7972.46	102	278
New Site	1248	0.686	1818.17	136	194
Yemoh Town	1858	0.404	4602.47	152	284
Njai Town	2298	0.216	10641.33	127	269
Kissi Town	2490	0.196	12709.81	154	287
Nduvuibu	2552	0.493	5177.21	205	343
New London	2873	0.597	4813.01	208	495
Kulanda Town	3882	0.294	13216.15	197	314
TOTAL	25,954	–	–	1979	4597

Residential and household survey data for 20 municipal sections of Bo, ordered by population, showing the persons per municipal section, section area, and the total number of residential and non-residential structures [2, 3]

The min, mid and max values

**Fig. 1:**
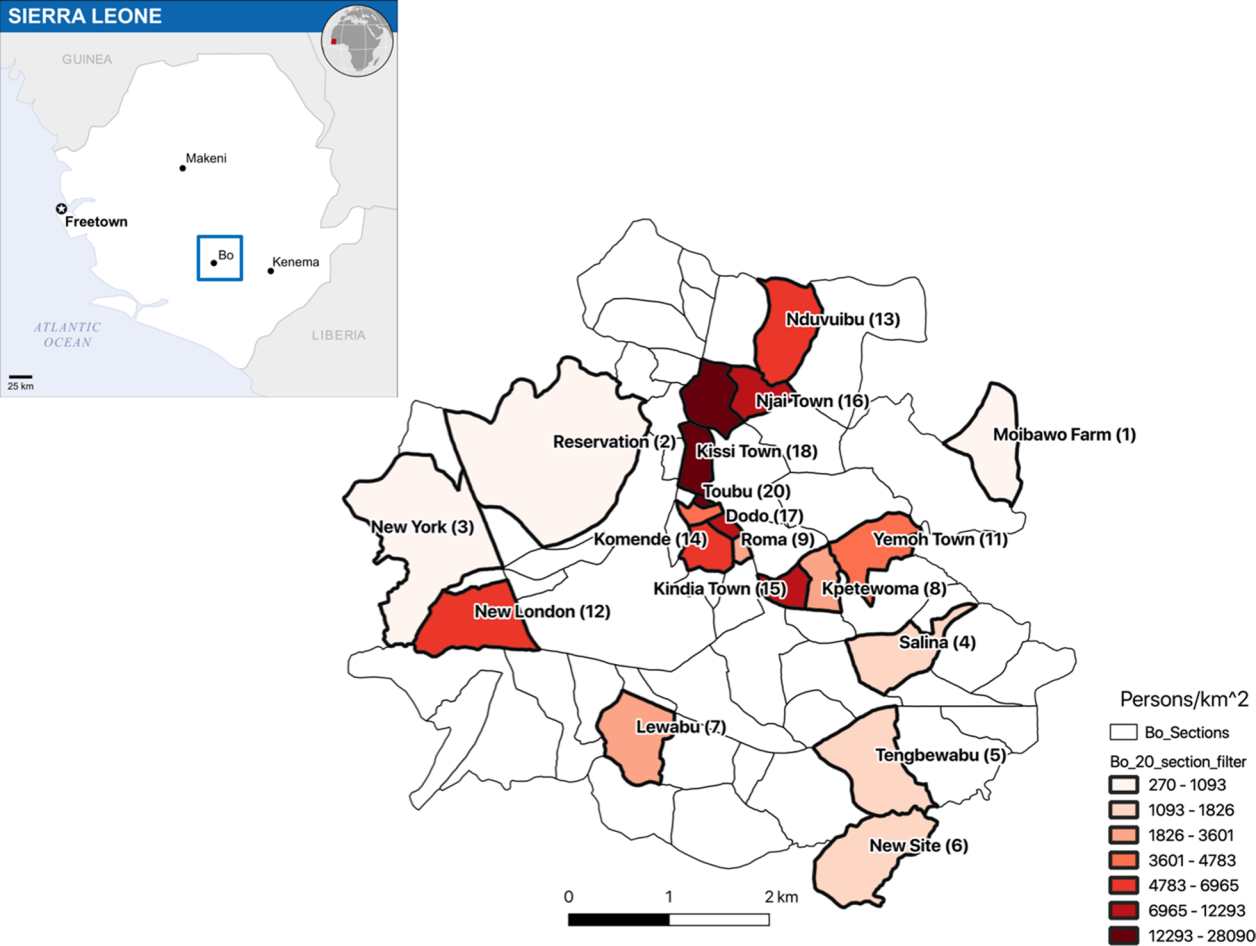
Bo sections ordered by population density. The 20 surveyed sections of Bo City ordered by population density $d = (persons)/km^2$. The inset in the upper left includes a map of Africa, with Sierra Leone highlighted in red and Bo City in blue (*Source*: OCHA/ReliefWeb). The larger map shows Bo City, the survey location

Our primary objective is to construct models for estimating the population densities ${\hat{d}}_{i = 1,\ldots ,20}$, from which estimates of the section populations ${\hat{p}}_{i = 1,\ldots ,20}$ will be derived. Fig 1 shows the 20 surveyed sections ordered by population density.

### Analytical approach

#### Estimating population densities

The use of satellite imagery for appraising land utilization, including population density estimation, is not novel. For a brief overview, see "Appendix 1". Our analysis uses selected TM measures of mean spectral reflectances (intensities), pixel-level spectral transforms, and diverse measures of spatial variability [that is, measures of *texture*] identified by Harvey [5] as candidate covariates. Because our population data are restricted to the measured populations of 20 sections in Bo, we test several different protocols for building and testing the regression models when sample sizes are small.

#### Estimating section populations

Given the estimated population densities for each section, the total population of the surveyed areas may be roughly estimated as the scalar product $\langle \,d,Area\rangle$ of the estimated population densities ${\widehat{d}}$ and the measured section areas. The implicit assumption is that the population density is relatively homogeneous within each section. This assumption is not satisfied for some of the sections surveyed, although the regression models developed are still surprisingly accurate.

#### Three questions to be addressed

After a preliminary discussion of materials and methods, we develop a regression model for estimating the population densities of the 20 sections. In particular, we address the following three questions:

1. Using the Landsat TM data to define a candidate set of independent variables, can we build one or more regression models for accurately estimating the measured population densities of the selected Bo City sections? The raw TM data consist of mean band-specific pixel-level intensity measurements for each section.
2. Can we then estimate the entire population of the sections in the dataset, given the estimated population densities of the individual sections, and the measured section areas?
3. Applying the $k-1$ cross-validation method (also referred to as “Leave one out cross-validation,” or LOOCV), how effectively do these regression models generalize to estimating the population density of a section deliberately omitted from the LOOCV training set?

## Methods and materials

### Survey methodology

The survey methodology is summarized in [3]. The data collection protocols for human subjects were approved by three independent Institutional Review Boards: Njala University, George Mason University, and the U.S. Naval Research Laboratory. Household data were collected from one adult representative of each participating household after obtaining written informed consent from that individual. Most residential structures were home to multiple households. To be defined as a resident of a household, a child or adult had to use the structure as sleeping quarters most nights. Family members who usually worked in other locations or attended boarding schools were not considered to be residents. The total population of each section was calculated by adding up the total number of residents in each residential structure. The data for the 20 surveyed sections listed in Table 1 have already been published in open-access literature.

### The Landsat thematic mapper (TM)

Landsat 5 was an Earth-observing satellite launched on March 1, 1984, into a near polar orbit at an altitude of 705 km, for collecting imagery of the Earth’s surface. It was decommissioned in January 2013. Landsat 5 instrumentation included a *Thematic Mapper* (TM) with an optical-mechanical “whisk broom” (along-track) scanner [6, 7]. The scanner’s mirror system bi-directionally swept the TM’s detectors along a line transverse to the north-south path of flight. The archived Landsat 5 TM scenes have an area of 170 km north-south by 183 km east-west (i.e. 106 mi by 114 mi). [8].

All data used in this article were derived from the scene LT52010542011001MPS01 [9] with the indicated path (201), row (54), date and year (2011/1/1). Publication of this imagery is in full compliance with guidelines [10, 11] authorizing the use and dissemination of USGS satellite imagery. The year 2011 was selected because the survey data for the population sections were collected in the same year [11]. Although Landsat 7 could have potentially provided more refined data, a failure of the TM scan line corrector (SLC) corrupted the scenes collected at the required dates (2011) and locations [12].

#### Correcting for atmospheric effects

The Landsat sensors capture reflected solar energy. The Landsat Ecosystem Disturbance Adaptive Processing System (LEDAPS) [13] is a software system for processing Landsat imagery to calculate the reflectance from the earth’s surface. A LEDAPS-processed dataset is available for the desired imagery [9]. The 3 major steps in LEDAPS processing are:

1. As a function of the band-specific sensor gain and bias, convert the Landsat sensor outputs to sensor spectral radiances, the energy reaching the sensors.
2. As a function of the earth-sun geometry and the mean solar exoatmospheric spectral irradiances, convert the spectral radiances to the *Top of the atmosphere* (TOA) dimensionless reflectances. The latter is the dimensionless ratio of reflected energy to total energy.
3. Estimate the reflected energy measured at the earth’s surface, rather than at the top of the atmosphere, by removing the interference imposed by the atmosphere itself on both the incoming and reflected solar radiation. This step requires correcting for wavelength-specific atmospheric scattering as well as masking and correcting for distortions imposed by cloud cover, shadows, and reflections from water.

#### TM data visualization

The TM data are multispectral, and each scene was captured in 7 different bands. Table 2 shows the bandwidth, resolution, and nominal utility for each of the 6 Landsat TM bands [14, 15] used in this study. The data from the different bands are usually combined to create complex images that enhance specific features of the target region.

**Table 2:** Landsat 5 thematic mapper bands used in this study

(1) Bands	(2) Wavelength ($\mu$m)	(3) Resolution (m)	(4) Applications
Band 1—blue	0.45–0.52	30	Because blue light is absorbed by chlorophyll, this band can be used to discriminate between vegetation, which will appear to be dark, and soil and/or roads and buildings, which are more reflective. Light in this band penetrates water deeply, which is useful for monitoring aquatic systems and for bathymetric mapping
Band 2—green	0.52–0.60	30	Green light is reflected strongly by chlorophyll, so this band is useful for appraising the state of vegetation. There will be high contrast between clear and turbid water, because light in this band penetrates water well, but with less scattering than in band 1
Band 3—red	0.63–0.69	30	This is the chlorophyll absorption band, and live vegetation will appear to be dark. This band is useful for differentiating between live plants with chlorophyll, which absorb red light, and dead foliage, which does not
Band 4—near infrared (NIR)	0.76–0.90	30	Useful for delineating shorelines, because water is strongly absorbent in this band, while soil and vegetation reflect brightly. It is a good band for differentiating soil from crops, and for quantifying biomasses
Band 5—shortwave infrared (SWIR) 1	1.55–1.75	30	This band is useful for measuring the moisture content of soil and vegetation. SWIR measurements can also help differentiate between snow on the ground, ice, and clouds in the air
Band 7—shortwave infrared (SWIR) 2	2.08–2.35	30	Similar in utility to Band 5, but also useful for differentiating between different types of rock formations

Landsat 5 thematic mapper band names, wavelengths, resolutions, and nominal domains. In this study, only measurements collected in Bands 1 through 5 and Band 7 were used. Band 6 measures thermal emissions, and was not used.

**Fig. 2:**
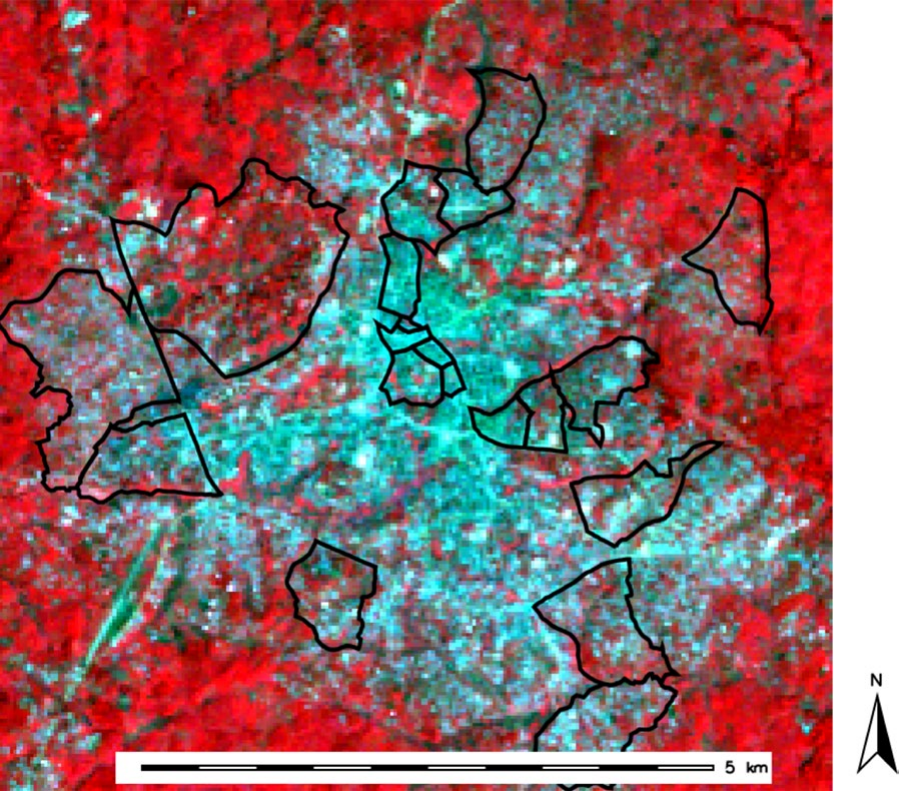
False-color near infrared (NIR) image of Bo city. Composite images are created by assigning the visible colors red (R), green (G), and blue (B) to TM greyscale bands [49]. The image shown is near infrared (NIR): [4,3,2]. Vegetation appears bright red in NIR images because near infrared band 4 is assigned to the color red, and chlorophyll is a good reflector of infrared

By mapping each band onto the visible colors red (R), green (G), and blue (B), the individual Bo City band images can be combined into different composite images [15]. The mappings are specified by indicating the sequence of bands assigned to the visible composite colors R, G, and B. In the “NIR” (near infrared) (bands 4, 3, and 2) mapping shown in Fig. 2, Band 4 is assigned to composite color R. Because vegetation reflects brightly in the NIR band 4, the vegetation surrounding Bo City appears to be bright red.

#### Pixel-level section representations

Six of the seven Landsat 5 TM bands were utilized. Band 6 in the TM sensor is emittance (temperature), and not normally used in combination with reflectance data; omitting Band 6, pixel-level matrix representations of the surface reflectance from each section can be made for each band using the LEDAPS corrected data.

**Table 3:**
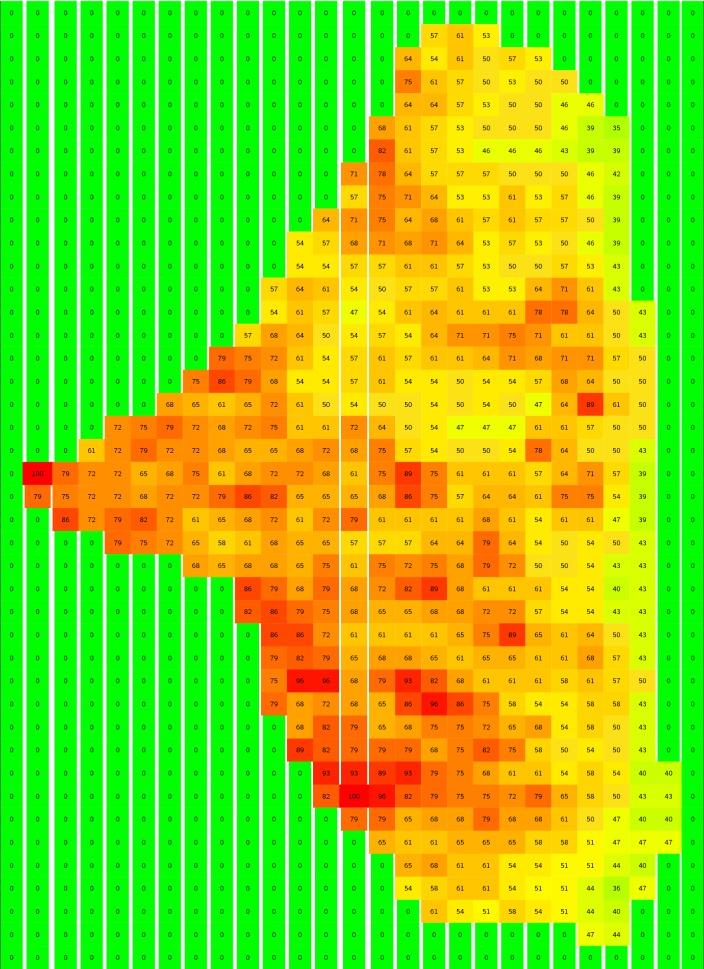
Moibawo normalized pixel amplitude distribution

Moibawo is approximately 0.5 km$^2$. In each of the 6 bands utilized, about 1000 pixels were scanned. This figure shows the magnitude and grid locations of the normalized pixel values (scaled from 0.0 to 100.0 for readability) measured in Band 3. Compare the shape of the non-zero elements in the above table to the outline of Moibawo in Fig. 1

**Table 4:**
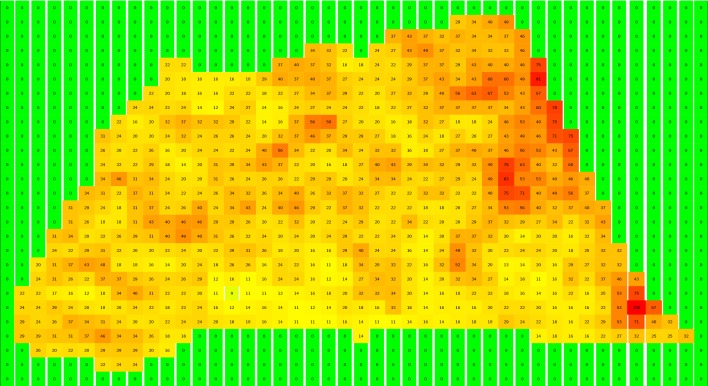
Moibawo normalized pixel amplitude distribution

New London section is approximately 0.5 km$^2$. In each of the 6 bands utilized, about 1050 pixels were scanned. This figure shows the magnitude and grid locations of the normalized pixel values (scaled from 0.0 to 100.0 for readability) measured in Band 3. The matrix of pixel values is rotated $90^{\circ }$ counterclockwise. Compare the shape of the non-zero elements in the above table to the outline of New London in Fig. 1

For example, the pixel magnitudes measured in Band 3 are shown in Tables 3 and 4 for Moibawo Farm and New London. The min-max normalization algorithm [16] was applied to rescale the sensor data between 0.0 and 1.0; in the two tables, these normalized values are multiplied by 100.0 to facilitate readability. Comparable visualizations could be made for each of the other bands. The area of New London is approximately 0.60 km$^2$, and Moibawo Farm is 0.50 km$^2$. The mean, standard deviation, and variance of the min-max pixel distributions defined the normalized variables *nb* [mean value of normalized LEDAPS-corrected pixel magnitudes], *nbs* [standard deviation], and *nbv* [variance] for these two sections in Band 3.

The resolution of the pixels for the 6 selected bands, including Band 3, is 30 *m*. There are 670 non-zero pixels in the New London section, and 559 pixels in the Moibawo Farm section. The areas estimated from these pixel distributions are consistent with the areas estimated from the shape files (i.e. map boundaries). Let $NP_i$ designate the number of pixels for each distribution, and *PA* the pixel area, which is always 900 $m^2$. The *i*th section $Area_i$ is then:

1 $$\begin{aligned} \mathbf {Area}_\mathbf {i}&=NP_{i}\times {PA}\times {10^{-6}}\frac{km^2}{m^2} \end{aligned}$$

The mean value of $nb_i$, the normalized pixels for Band *i*, is:

2 $$\begin{aligned} \mathbf {nb}_\mathbf {i}&= {\frac{1}{\mathbf {NP}_{\mathbf {i}}}{\sum _{\mathbf {n=1}}^{\mathbf {NP}}\mathbf {b}_{\mathbf {3}_{\mathbf {n}}}}} \end{aligned}$$

The variances and standard deviations for these distributions can be similarly derived.

### Estimation methods

The premise of this paper is that low-dimensional subsets of variables derived from Landsat data can be used to construct accurate regression equations for estimating the population densities of the 20 surveyed sections. In this section, we will describe the datasets, methods, and metrics that were used. Figure 1 is a color encoded map showing the population densities of the 20 surveyed sections.

#### The TM covariate dataset

In his study [5], Harvey proposed a large set of candidate Landsat TM covariates for estimating population densities in Australian census districts. He reduced this preliminary set of variables to a low-order set of covariates through a complex sequence of model testing.

We used Harvey’s full set of proposed candidate variables for our regression analysis. An obvious objection is that Harvey’s models were tailored to estimate population densities in the urban northern latitudes of Australia, whereas we were fitting our models to estimate population densities in a region where much of the population resides in informal settlements. However, we utilized the full instantiated set of candidate variables, with some exceptions to be noted, as input to our model selection algorithms. At no point did we use the reduced sets of candidate variables or the specific regression models that were trained and tested against Australian census data. The regression covariates selected during model construction therefore reflected the unique attributes of our Bo City dataset.

Our methodology also used improved methods. Rather than implementing the TOA and atmospheric corrections manually, as Harvey was required to do given the technical restraints at the time, we were able to use the LEDAPS-processed imagery provided by USGS. We also used Bayesian MCMC (Markov chain Monte Carlo) sampling to find the variables for our regression models, rather than step-wise regression, although the latter remains a viable approach.

#### TM variable definitions

Multiple candidate variables were calculated for each of the 20 Bo City sections. To simplify the notation, the index term for the section [i.e. a number between 1 and 20] has been omitted, as there are no variables that are functions of more than one section. See Table 5.

**Table 5:** Landsat 5 thematic mapper candidate covariates

Variables	Covariate subset list	Variable definition	Number of variables	Non-spectral mean values	Spectral (pixel-level) transform
	*Non-spectral transforms*				
B	$b_i$; i = 1, 2, 3, 4, 5, 7	Mean value of Landsat 5 thematic mapper $\hbox {band}_i$ measurement	6	*	
Bs	$bs_i$ ...	SD of $b_i$	6	*	
Bv	$bv_i$ ...	Variance of $b_i$	6	*	
Bc	$bc_i$ ...	Coefficent of Variation [CV]: $bc_i$ = sd($b_i$)/$b_i$ = $bs_i$/$b_i$	6	*	
S	$s_i$; i = 1, 2, 3, 4, 5, 7	Square of $b_i$ = $b_i$ x $b_i$	6	*	
P	$p_{ij}$; i = 1, 2, 3, 4, 5 and j = i + 1, ..., 5, 7	Non-spectral cross products of the means $b_i$ and $b_j$	15	*	
R.re	$r.re_{ij}$; i = 1, 2, 3, 4, 5 and j = i + 1, ... , 5, 7	Non-spectral ratios of means $b_i$ and $b_j$ = $b_i$/$b_j$	15	*	
D	$d_{12}$; i = 1, 2, 3, 4, 5 and j = i + 1 , ... , 5, 7	Non-spectral ratio of the difference-to-the-sum of the mean value $b_i$, $b_j$: $d_{ij}$= ($b_i$-$b_j$)/($b_i$+$b_j$). See text	15	*	
	*Spectral transforms*				
NB	$nb_i$; i = 1, 2, 3, 4, 5, 7	Mean value of the min-max normalized $\hbox {band}_i$ measurements (see text)	6		*
NBs	$nbs_1$ ...	SD of $nb_i$	6		*
NBv	$nbv_i$ ...	Variance of $nb_i$	6		*
NBc	$nbc_i$ ...	Coefficent of Variation [CV]: $nbc_i$= sd($nb_i$)/$nb_i$ = $nbs_i$/$nb_i$	6		*
R	$r_{ij}$; i = 1, 2, 3, 4, 5 and j = i + 1, ... , 5, 7	$r_{ij}$ = mean ratio of the paired pixel magnitudes	15		*
Rs	$rs_{ij}$ ...	SD of $r_{ij}$	15		*
Rv	$rv_{ij}$ ...	variance of $r_{ij}$	15		*
Rc	$rc_{ij}$ ...	Coefficient of Variance [CV] of $r_{ij}$	15		*
DS	$ds_{ij}$; i = 1, 2, 3, 4, 5 and j = i + 1, ... , 5, 7	$ds_{ij}$ = mean ratio of the difference-to–sum of the paired pixel magnitudes	15		*
DSs	$ds_{ij}s$ ...	$ds_{ij}s$ = SD of $ds_{ij}$	15		*
DSv	$ds_{ij}v$ ...	$ds_{ij}s$ = variance of $ds_{ij}$	15		*
DSc	$ds_{ij}c$ ...	$ds_{ij}s$ = coefficient of variation [CV] of $ds_{ij}$	15		*
CH	$ch_{ijk}$; i = 1, 2, 3, 4; j = i + 1, ... , 5; k = j + 1	Cylindrical transform of composite $hue_{ijk}$ - see text	20		*
CHs	$ch_{ijk}s$ ...	$ch_{ijk}s$ = SD of $ch_{ijk}$	20		*
CHv	$ch_{ijk}v$ ...	$ch_{ijk}v$ = variance of $ch_{ijk}$	20		*
CHc	$ch_{ijk}c$ ...	$ch_{ijk}c$ = coefficient of variation [CV] of $ch_{ij}$	20		*
RH	$rh_{ijk}$; i = 1, 2, 3, 4; j = i + 1, ... ,5; k = j + 1	Rectangular transform transform of composite $hue_{ijk}$ (see text)	20		*
RHs	$rh_{ijk}s$ ...	$rh_{ijk}s$ = SD of $rh_{ijk}$	20		*
RHv	*rh* *ijkv* ...	$rh_{ijk}v$ = variance of $rh_{ijk}$	20		*
RHc	$rh_{ijk}c$ ...	$rh_{ijk}c$ = coefficient of variation [CV] of $rh_{ij}$	20		*
		Total variables:	379	75	304

A summary of the 379 Landsat 5 thematic mapper variables calculated for this study. Only measurements collected in Bands 1 through 5 and Band 7 are used

Let *p* denote the number of pixels sampled in a given section and $b_{i_n}$ denote the value of the Landsat thematic mapper (TM) sensor measurement of the *n*th pixel in band *i*. For each pixel, measurements were made in bands 1,2,3,4,5 and 7; *i* is restricted to these values. Additional candidate covariates were then derived from the LEDAPS-corrected pixel-level intensity measurements. Table 5 summarizes the 3 datasets used in subsequent analysis: (1) non-spectral transforms, (2) spectral transforms, and (3) the total combined dataset. There are 379 total variables, with a subset of 304 spectral transforms and 75 non-spectral transforms. The definitions and equations for all variables in Table 5 are given in "Appendix 2". The initial set of 379 candidate covariates was substantially reduced prior to initiating the regression analysis per se, using methods described below.

#### The TM data array

The 20 measured observations of persons per section, in combination with the measured section areas, yield the dependent variables $d_i=\frac{Persons_i}{Area_{i = 1,\ldots 20}}$. Our model estimates $d_i$ as a function of the Landsat TM measurements. The Landsat Thematic Mapper (TM) measurements and derived variables can be arranged in an array with 20 rows and 379 columns. Each row denotes a Bo City section, and each column corresponds to one of the 379 variables derived from the Landsat TM data. This array is shown schematically in Table 6. Two columns of demographic variables (*section name* and $d = {population\,density}$) precede the 379 columns of TM data.

**Table 6:** Bo municipal survey population density data and abbreviated Landsat band data, tabulated by section

(1) Section	(2) Persons/km^2^	(3) b1	(4) b1c	(5) –	(6)[col 380] s5	(7) [col. 381] s7
Moibawo farm	135	0.04988	0.11564	–	0.04902	0.01545
Roma	139	0.0661	0.06879	–	0.0497	0.02889
Bo Central	273	0.07276	0.09094	–	0.04855	0.03639
Toubu	454	0.0657	0.11885	–	0.04008	0.02383
Salina	580	0.05845	0.14618	–	0.05418	0.02302
Dodo	597	0.06779	0.06786	–	0.04359	0.03019
Reservation	637	0.05072	0.13245	–	0.0442	0.01411
Kpetewoma	640	0.05775	0.10814	–	0.04936	0.0209
Lewabu	879	0.05604	0.14217	–	0.04967	0.01897
Tengbewabu	1068	0.05798	0.13686	–	0.04854	0.01896
New York	1088	0.05675	0.1109	–	0.05054	0.01932
Komende	1103	0.06226	0.10095	–	0.04478	0.02323
Kindia Town	1160	0.05992	0.07469	–	0.04318	0.0223
New Site	1248	0.05861	0.14711	–	0.05713	0.02422
Yemoh Town	1858	0.05627	0.1299	–	0.03849	0.01577
Njai Town	2298	0.0587	0.10871	–	0.04057	0.01859
Kissi Town	2490	0.06258	0.08847	–	0.04161	0.02462
Nduvuibu	2552	0.05408	0.10372	–	0.03665	0.01464
New London	2873	0.05782	0.12842	–	0.04221	0.01777
Kulanda Town	3882	0.05779	0.11445	–	0.04022	0.01746

Table entries are ordered by population. The section (municipality) name is in column 1, and the population density *d *= persons/km$^2$ is in column 2. See [2, 3]. Appended to the first 2 columns are representative Landsat band filter measurements 1–2 and 378–379 (see text). The measured number of persons/km$^2$ in the *i*th section (i.e. $d_i$) is the dependent variable to be estimated

### Regression models

#### Software development

The regression simulations and auxiliary plotting functions were written in the programming language R by the first author. Support functions from multiple R libraries were used, particularly [17]. The second author developed additional R code for processing the Landsat imagery, and produced the 20 by 379 matrix of Landsat TM derived products.

#### Regression methods

We will now summarize the major steps:

1. Data reduction. We began with a data array containing 379 candidate regression covariates. This was reduced to an array of 159 covariates prior to conducting the regression analysis. First, the subset of 304 spectral transforms alone was found to yield a good solution. Second, if the Pearson correlation between a pair of covariates was .99 or greater, one of the covariates was dropped.
2. Data transformation. Different candidate transforms for the dependent variable $d_{i}=persons_i/km^2$ were evaluated to improve the linearity of the regressive estimator for ${\hat{d}}$. The square root transform $\sqrt{d}$ was selected as the dependent variable to be estimated.
3. Regression analysis. A Bayesian mixture analysis was run, using an MCMC *(Markov chain Monte Carlo)* Metropolis-Hastings sampler to evaluate the candidate regression equations [17–19]. A brief summary of the methods used is provided in Appendix 3. The best single equation found for estimating $\widehat{\sqrt{d}}$ during the stochastic sampling was transformed to a conventional linear multiple regression equation.
4. Back-transform $\widehat{\sqrt{d}}$. The transformed estimated population density vector $\widehat{\sqrt{d}}$ was back-transformed [20] into the original parameter space as $\widehat{d_i}$. The goodness-of-fit of the regression equation for estimating ${\widehat{d}}$ could then be evaluated. The population of each section was also estimated.
5. Cross-validation. “Leave-out one cross-validation” (LOOCV) [21] was used to quantify how well the regression equation generalizes to estimating observations that were not included in the training set.

## Results

### Data reduction

The original Landsat data array has 379 candidate regression covariates. Reducing the size of this dataset should increase the effectiveness of the MCMC sampling algorithm by reducing the size of the regression model search space. PCA (Principal Components Analysis) is often used to reduce a large dataset prior to subsequent analysis, but PCA transforms the original variable set by mapping combinations of variables onto a new coordinate system. We wanted to identify the *individual* Landsat variables which were most critical for estimating the population density, so PCA was not an appropriate method.

Two preliminary steps were used to reduce the dataset prior to MCMC sampling. First, by trial-and-error we found that all of the covariates selected were from the subset of Landsat variables defined for spectral (i.e. inter-pixel) transforms (Table 5). Using only the spectral transform subset of variables reduced the size of the data array from 379 candidate covariates to 304 candidate covariates. Second, we removed a member of each pair of “identical” covariates whose Pearson correlation was 0.99 or greater [22]. The set of 304 covariates was reduced to a set of 159 covariates without any degradation on the quality of the regression models. See Table 7.

**Table 7:** This table summarizes the number of candidate covariates retained at each stage of model development

(1) Step	(2) Number of covariates
Initial data set	379
Use spectral transforms only	304
Remove redundant highly-correlated variables	159
MCMC best regression model	6

### Data transformation

**Fig. 3:**
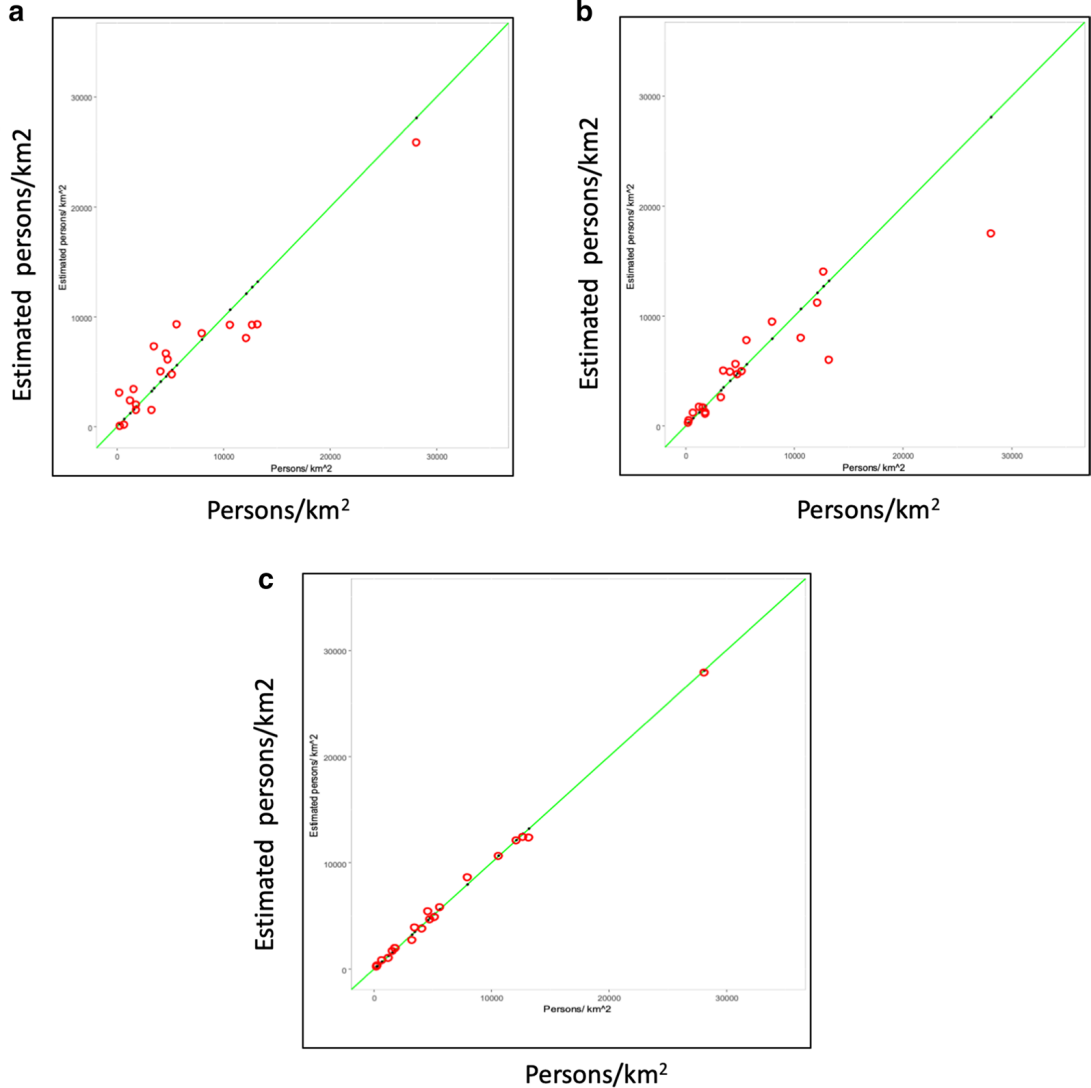
Back-transformed estimators of the $ln(d_i)$ and $\sqrt{d_i}$ of the population density $d_i$. (**a**) No transform (**b**) back-transform of $\widehat{ln{(d_i)}}=e^{\widehat{ln(d_i)}}$ (**c**) back-transform of $\widehat{\sqrt{d_i}}={[\widehat{\sqrt{d_i}}}]^2$

Figure 3 shows the back-transformed estimated population density for ($\hat{d_i}$ = persons$_i$/km$^2$), plotted as a function of the section population density for each transform of *d*. The regression model used was the top model in an ordered mixture of the 1000 best-fitting regressions found in the MCMC sample space. The green line is the true value of *d*. No transform was applied in plot (A), (B) is the back-transformed log transform (i.e. $e^{\widehat{ln(d)}}$), and (C) is the back-transformed square root transform (i.e. $[{\widehat{\sqrt{d}}}]^2$). The square root transform ${\widehat{\sqrt{d}}}$ yielded the most linear estimation of the population density.

### Regression analysis

Table 8 gives the parameters for the best regression model found for estimating $\sqrt{d}$ using the sampling protocol summarized in Appendix 3. Given the low values of the VIF, there is no significant multicollinearity between the selected variables (col 7). The fit of the model is excellent: $R^{2}=.9951$ and $R^{2}.\,adjusted = 0.9928$, on 6 and 13 degrees of freedom. See Table 9. The regression was run on the transformed population density variable (i.e. on the square root of the population density). The square root transform generated a more linear relationship between the Landsat sensor readings and the dependent variable of section population than a log transform or no transform, which contributes to the high values of $R^{2}$ for the transformed variable. See Fig. 3 for a comparison of regression plots made using log and square root transforms and no transform at all.

**Table 8:** The best regression model found by the MCMC sampler

(1) Covariates	(2) Estimated coefficient	(3) Std. Error	(4) t value	(5) Pr($>|t|$)	(6) Max. Prob.	(7) VIF	(8) PIP
(intercept)	− 174.02	20.16	− 8.63	9.63E−7	< .0001	–	–
nb7v	− 1656.91	85.58	− 19.36	5.72E−11	< .0001	6.29	0.9837
r_sp37	532.23	31.27	17.02	2.88E−10	< .0001	1.34	0.9790
nb1v	1686.14	71.28	23.66	4.52E−12	< .0001	5.36	0.9500
r_sp15s	− 2744.29	159.21	− 17.24	2.46E−10	< .0001	1.08	0.4711
ch245c	44775.32	2636.73	16.98	2.96E−10	< .0001	2.72	0.9835
r_sp14c	− 246.75	23.79	− 10.37	1.18E−07	< .0001	1.55	0.5381

This table summarizes the best regression equation returned by the MCMC sampler for the estimation of $\sqrt{d}$. The values of the variance inflation factor (VIF) are less than 7.0, which demonstrates the low collinearity between the covariates. Four of Posterior Inclusion Probabilities (PIPs) are close to 1.0, quantifying their importance as predictive variables of $\sqrt{d}$, as discussed in the text

**Table 9:** Measured and estimated values of population and population density

(1) Name	2) p = Population	(3) Measured area in km$^2$	(4) d = Persons per km$^2$	(5) Estimated ${\hat{d}}$	(6) Estimated ${\hat{p}}$	(7)* RE* (%)
Moibawo Farm	135	0.5	270	206.13	103	− 24
Roma	139	0.04	3475	3818.48	153	10
Bo Central	273	0.066	4136.36	3753.27	248	− 9
Toubu	454	0.016	28375	27852.13	446	− 2
Salina	580	0.467	1241.97	1164.25	544	− 6
Dodo	597	0.049	12183.67	12234.85	600	0
Reservation	637	2.329	273.51	282.25	657	3
Kpetewoma	640	0.197	3248.73	2918.91	575	− 10
Lewabu	879	0.479	1835.07	2100.07	1006	14
Tengbewabu	1068	0.68	1570.59	1770.75	1204	13
New York	1088	1.513	719.1	585.41	886	− 19
Komende	1103	0.196	5627.55	5780.18	1133	3
Kindia Town	1160	0.146	7945.21	8806.6	1286	11
New Site	1248	0.686	1819.24	2061.33	1414	13
Yemoh Town	1858	0.404	4599.01	5379.96	2174	17
Njai Town	2298	0.216	10638.89	10524.21	2273	− 1
Kissi Town	2490	0.196	12704.08	12479.6	2446	− 2
Nduvuibu	2552	0.493	5176.47	4803.58	2368	− 7
New London	2873	0.597	4812.4	4487.98	2679	− 7
Kulanda Town	3882	0.294	13204.08	12452.93	3661	− 6

This table lists the (1) section name (2) measured section population (3) measured section area in $km^2$ (4) population density $d = Persons/km^2$ (5) the regression-estimated population density ${\hat{d}}$ (6) the estimated population/section $\hat{p_{i}}=Area*{\hat{d}}$. (7) the % Relative Error (*RE*) for the density estimation

One indication that a good solution has been found in the sample space is that the MCMC sampler frequencies and the analytical posterior marginal likelihoods both converged. For $10^7$ iterations, the correlations were almost perfect (0.9657) between the empirical and analytical distributions.

### Estimating section areas

**Fig. 4:**
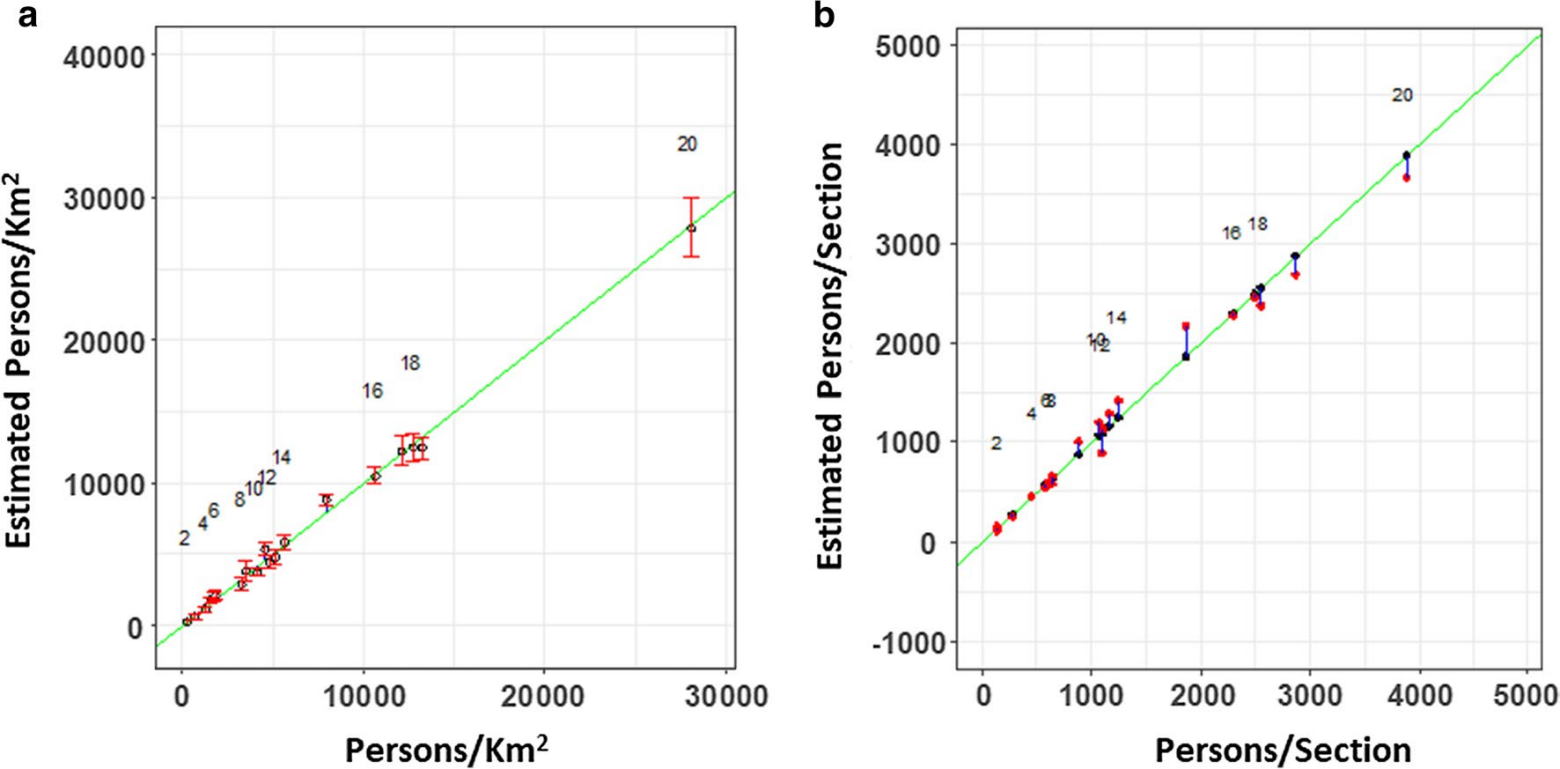
Back-transformed regressive estimations of ${\hat{d}}_i$ and ${\hat{p}}_i$. (**a**) The back-transformed estimates of the square root of the population density $\hat{d_i}={[\widehat{\sqrt{d_i}}}]^2$, shown with back-transformed 95% confidence intervals. (**b**) The estimated section populations

Figure 4a shows the back-transformed estimates of the populations densities ${\widehat{d}}_i$, plotted as a function of the measured population densities. The regression equation in Table 8 was used to estimate $\widehat{\sqrt{d}}$. The vector of estimates, and their .95 confidence intervals, were both back-transformed into the original parameter space: ${\hat{d}}=[\widehat{\sqrt{d}}]^2$ [20]. Panel (B) shows the estimate of the population obtained by multiplying the back-transformed estimate of ${\hat{d}}$ by the measured section areas:

3 $$\begin{aligned} \begin{aligned} {\hat{{\mathbf {p}}_{\mathbf {i}}}}&= Area_i\times \hat{d_i} \end{aligned} \end{aligned}$$

### *Relative proportional errorRE*

Harvey [5] recommends the *Relative* or *Proportional Error* as a measure of fit, rather than $R^2$, and we will provide these values for the regression error.The *Relative Error*, which will be abbreviated here as the *RE*), is defined as:

4 $$\begin{aligned} \mathbf {RE}=\frac{{\hat{\mathbf{d }}}_\mathbf{i }-{\mathbf{d }_\mathbf{i }}}{\mathbf{d }_ \mathbf{i }}\times \mathbf {100}\% \end{aligned}$$

This measure is the same for both the population and the population density. It can be calculated for the estimated transform of the population density $\widehat{\sqrt{d}}$ and the estimated *back-transformed* population density ${\hat{d}}={[\widehat{\sqrt{(}d)}}]^2$. The *RE* can be positive or negative, and the Mean *RE* is the mean of the absolute values of *RE*.

**Fig. 5:**
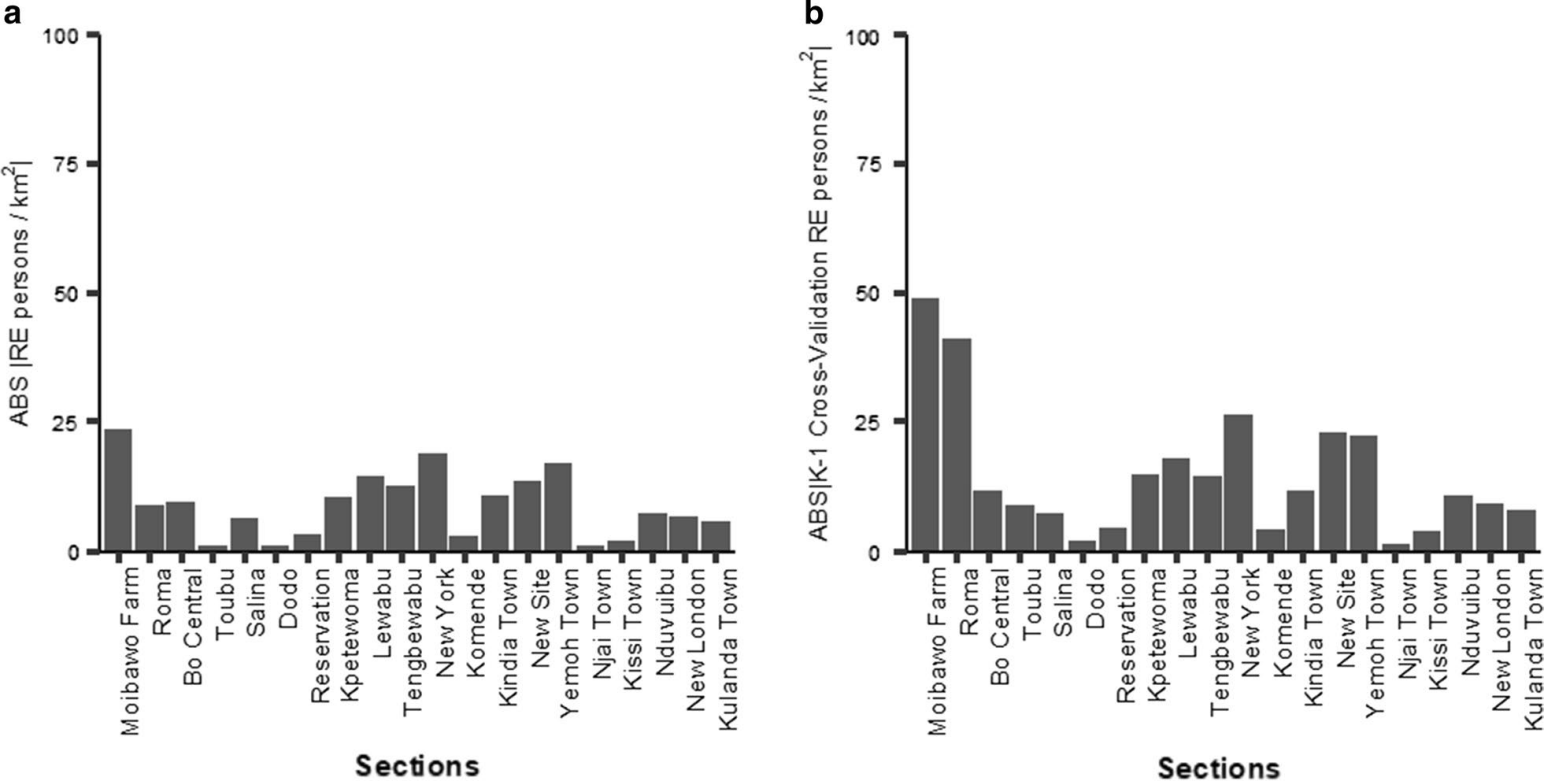
The Relative Errors *REs* for the back-transformed estimate ${\hat{d}}_i$ and cross-validation trials. (**a**) The absolute value of the *RE* (*% Relative Error*) for the back-transformed estimate $\hat{d_i}={[\widehat{\sqrt{(}d_i)}}]^2$ (**b**) The absolute value of the *RE* for the 20 “Leave-one-out cross-validation” (LOOCV) trials

Table 9 lists the *REs* by section, as well as $\hat{d_i}$ and $\hat{p_i}$. The absolute value of the *RE* is shown in Fig 5. The fit is very good, and the median absolute *RE* is 8.0%. The *REs* for all sections is less than 20%, with the exception of Moibawo Farm, where the population density is underestimated by 24%.

### *LOOCV* cross-validation

In our current study, the number of aggregated population observations is 20. This is insufficient to divide the observations into training groups (sets) and test groups (sets), as is normally done for cross-validation. As an alternative, we used k-1 cross-validation, where $k=20$. Assume that a regression equation with *x* independent variables has been found for estimating $d_i$ for all *k* observations, where each observation is the measured population density $d_i$. Referring to Table 8, $x=6$ and $n=20$. There are *k* *LOOCV* (Leave-Out One Cross-Validation) tests that can be constructed and executed. An obvious drawback is that there is only a single observation available for estimation on each trial.

In each of the *n* *LOOCV* trials, a single observation $d_j$ was omitted from the dataset. Using the same *x* independent variables, a new regression model was fitted to the remaining $n-1$ population density observations $d_i$. The reduced model was then used to estimate the single omitted population density $d_j$. This process was repeated for all *n* trials. A different regression equation was parameterized for each of the *n* trials, but the same set of *x* independent variables was always used. Figure 5 shows the absolute value of the percentage relative error *RE* for each section. Table 10 shows the details of the calculation, as well as the *RE* for the transformed population density $\sqrt{d}$. In Fig. 5, the bar charts show both the relative error (*RE*) for the estimation of the population density by section and the absolute values of the *RE* for the cross-validation tests.

**Table 10:** This table summarizes the results from the k − 1 ’LOOCV’ cross-validation analysis

Name	(1)	(2)	(3)	(4)	(5)	(6)	(7)	(8)	(9)	(10)
	*Pop*	*Area*	*d*	$\sqrt{(}d)$	${\widehat{d}}$	$\widehat{d^2}$	$Er(\sqrt{d})$	$\%{Er(\sqrt{d}}$	$Er(\widehat{d^2})$	$\%{RE(\widehat{d^2})}$
Roma	139	0.04	3510.72	59.25	70.39	4954.9	11.14	18.8	1444.18	41.14
Bo Central	273	0.066	4137.9	64.33	60.44	3653.53	3.88	6.03	484.37	− 11.71
Toubu	454	0.016	28089.66	167.6	160.05	25617.12	7.55	4.5	2472.54	− 8.80
Salina	580	0.467	1242.8	35.25	33.94	1151.93	1.31	3.73	90.87	− 7.31
Dodo	597	0.049	12126.22	110.12	111.22	12370.87	1.11	1	244.65	2.02
Reservation	637	2.329	273.5	16.54	16.91	285.8	0.37	2.22	12.3	4.50
Kpetewoma	640	0.197	3250.74	57.02	52.63	2769.82	4.39	7.69	480.92	− 14.79
Lewabu	879	0.479	1836.16	42.85	46.54	2166.06	3.69	8.61	329.9	17.97
Tengbewabu	1068	0.68	1571.17	39.64	42.41	1798.53	2.77	6.99	227.36	14.47
New York	1088	1.513	719.3	26.82	23.01	529.41	3.81	14.21	189.89	− 26.40
Komende	1103	0.196	5622.16	74.98	76.52	5856.04	1.54	2.06	233.88	4.16
Kindia Town	1160	0.146	7972.46	89.29	94.36	8903.89	5.07	5.68	931.43	11.68
New Site	1248	0.686	1818.17	42.64	47.27	2234.43	4.63	10.86	416.26	22.89
Yemoh Town	1858	0.404	4602.47	67.84	75.01	5626.04	7.17	10.56	1023.57	22.24
Median (abs)	1078.00	0.35	4370.19	66.09	67.11	4504.31	3.85	5.57	424.25	11.15
Mean (abs)	1297.70	0.48	6180.05	69.14	69.00	6098.19	3.89	7.41	568.21	14.58

For each section, col (1) is the section population, Col (2) is the area in $km^2$, and column (3) is the population density $d = persons/area$. Col (4) is the transform $\sqrt{d}$, col (5) is the estimate $\widehat{\sqrt{d}}$, and (6) is the back-transformed estimate of ${\hat{d}}$. Cols (7) and (8) are the error (*Er*) and *RE*) of the estimated variable $\widehat{\sqrt{d}}$; and cols (9) and (10) are the corresponding error functions for the back-transformed estimate of *d*

Although the median absolute value of *RE* for the back-transformed estimate is only 11.14%, the model failed to generalize (i.e. cross-validate) well in at least 3 cases. *d* for Moibawo Farm was underestimated by almost 50%, New York was underestimated by over 26%, and Roma was overestimated by about 41%. It is difficult to discern a simple pattern in the outliers. Moibawo Farm, like Reservation, has large open non-residential areas. But if this caused the underestimation in population density, the estimate for Reservation should have been similarly affected.

## Discussion

The model used in our research was specific to the 20 sections that we studied. The cross-validation study demonstrates that the six covariates in the regression model could be used to construct 19 separate regression equations for estimating the population density *d* of an omitted section, although there were several outliers noted. The model has not yet been tested in other urban areas with different patterns of residential structures, building materials, roads, or other characteristics, and it is likely that adaptation to the model and variables would be required.

Because the MCMC sampling of the solution space is stochastic and incomplete, the regression model summarized in Table 8 is not unique, although the “top model” solution was very effective for predicting *d*. A fixed random number seed was used in the simulations to enable the replication of results between simulations. Given different initial random number seeds, or alternative numbers of sampler iterations, alternate solutions could have been found.

All six of the selected regression variables are measures of covariate spatial variation (variance, coefficient of variation, and standard deviation), as can be seen in Table 8. These measures denote spatial variations in brightness between relatively large 30 *m* pixels. A typical Bo residential structure is smaller than a single 30 *m* pixel, and these measures of spatial variation cannot capture fine-scale modulations in reflectance within individual rooftops. The TM resolution is also insufficient for the application of feature extraction algorithms for explicit capture of rooftops or other structural boundaries [23, 24].

### Statistical significance of individual regressors

The stochastic nature of the simulation does not, however, diminish the significance of the variables selected with respect to their relative importance in the sample space as good candidate predictive variables (i.e. regression covariates) for estimating *d*. Four of the PIP (*posterior inclusion probability*)values are were close to 1.0. It is highly likely they would be included in *any* of the 1000 best-fitting models that were retained by the sampler, as well as in the “top” model. (The number of top models tracked by MCMC sampler is user-selectable.) Four covariates (*nb*7*v*, $r\_sp37$, *nb*1*v*, and *ch*245*c*) out of the six in the regression equation have PIPs close to 1.0. The high PIP values indicated that all four variables were included in almost every one of the 1000 best-fitting models tracked by the Bayesian MCMC sampler, which implies that the selection of these four variables was robust. The PIPs of the remaining two covariates were 0.47 and 0.54; each was retained in about half of the 1000 best regression models. The PIP is also proportional to Schwarz’s *Bayesian information criterion* (BIC) [25, 26].

Another advantage of our approach is that each of the six regression covariates was calculated directly from Landsat imagery, rather than as a transform of multiple Landsat variables. In data reduction methods such as PCA (Principle Components Analysis), the significance of the individual Landsat variables may be obscured by the complex mapping of the individual variables into the transform space.

### Interpreting spectral signatures

The variables and combinations of variables that were selected for the regression model are consistent with our understanding of the natural world. Within this scene, one can see that the unpopulated areas are heavily vegetated whereas the populated areas surveyed are a combination of tarpaulin and zinc/aluminum roofs, paved and unpaved driving/walking surfaces, as well as bare earth and vegetation between structures. The interpretation of why specific combinations of variables were selected is somewhat conjectural.

With the exception of $r\_sp37$, all of the covariates are measures of spatial variation (“texture”), rather than measures of brightness. For the band 7 covariate *nb*7*v*, a high variance is negatively associated with *d*; this band can aid in the differentiation between soil types and minerals, and is also sensitive to water content. *ch*245*c* is the coefficient of variation (CV) for a cylindrical transform of bands 2, 4, and 5; this tri-band mapping onto a single value constitutes a form of data compression. All 3 bands reflect vegetation brightly, but it is the CV that appears to be positively associated with the population density.

A characteristic of regional statistics, like the ones we used, is that each region has different fractional amounts of the previously stated ground cover materials. Man-made materials often reflect more in the infrared portion of the spectra (e.g. NIR, SWIR1, and SWIR2) as compared to vegetation, and vegetation absorbs more light in the visible portion of the spectra (e.g. blue, green, red) as compared to soil and man-made materials. Armed with this knowledge, we can infer that the multiple variables used in the regression analysis are differentiating the natural, vegetated areas from the built up regions to deduce population density in the region.

The inclusion of the blue band is present in three of the variables: *nb*1*v*, $r\_sp15s$, and $r\_sp14c$ in Table 8. This seems noteworthy, given the interaction between blue light and Rayleigh scattering as well as Mie scattering. Particulates of various sizes in the atmosphere can either selectively scatter shorter wavelengths (e.g. blue and violet via Rayleigh scattering) or scatter light over a broader wavelength range (e.g. Mie scattering). As part of our future research, we would like to examine how blue light is scattered as a result of particulates in the atmosphere over urban areas as compared to that of densely forested areas, and to see if this is a critical factor for interpreting spectral signatures.

### Correcting for non-homogeneous population density

An implicit assumption of this approach is that the population density is relatively homogeneous within a section. This assumption can be problematic in at least 3 ways:

1. If an area (section) is primarily wild vegetation or barren soil, it violates the assumption that the population density is relatively uniform within an area. If so, the spectral statistics for a section may primarily be a function of an “empty” region on the ground, rather than being representative of an area populated (although perhaps sparsely) with built structures and associated property. The Bo City section Reservation provides an extreme example of both issues. This section is essentially a large swamp, with a small number of buildings at the perimeter [3] that were originally constructed for government use.
2. A predominance of non-residential buildings within an area may confound residential and non-residential regions. Other than collecting additional survey data, or utilizing local knowledge to annotate the section imagery, there is no obvious way to differentiate between residential and non-residential structures in the Landsat imagery.
3. Variation in rooftop materials can confound the sensor interpretation within a given area. However, in the 20 sections surveyed, we would not expect great variation in sensor readings attributable to differences in roofing materials. Of the 1165 residential structures surveyed in the 20 sections, 1156 had zinc roofs, 8 had tarpaulins, and one was “other.” For this reason, it is unlikely that we confounded residential rooftops with bare earth or cultivated land.

### *Land-use/land-cover* models

(LU/LC) modeling offers another approach to resolving the consequences of non-homogeneous land use. A LU/LC model would differentiate between categories of land use in different areas of a section, distinguish between residential and non-residential structures, and allow for differences in rooftop construction. Wilson and his collaborators have developed LU/LC models for Bo City that dramatically illustrate the changes in LU/LC as consequence of forced migration during civil conflict between 1998 and 2002. For example, in [27] see Figures 7 and 8, and the accompanying tables. Although elegant, this approach would require a level of ground-truth data, data fusion, and model development that cannot be achieved within the scope of our approach.

### Spatial autocorrelation and image resolution

Spatial autocorrelation methods [28] are not new, but the power of these statistical methods has been enhanced by the advent of high-speed computers, the availability of large GIS datasets [29], and the development of custom software packages that facilitate the work of the analyst [30]. The interactions between spatial entities are usually modeled as a function of adjacency (i.e. contiguities between polygonal representations) and/or distance. The links denoting distance can also be weighted. Both global (e.g. Moran’s *I*) and local (e.g. LISA, *Local Indicators of Spatial Association*; Geary’s *c*) measures of spatial autocorrelation have been developed [28, 30].

There is a significant interaction between spatial autocorrelation patterns and map resolution [31]. As a concise example, Spiker and Warner [32] derived autocorrelation measures for a satellite image of Morgantown, WV, at three different pixel sizes: 0.7m, 15m, and 60m. The local value of Moran’s *I* is sensitive to buildings and other features of the urban infrastructure at high resolution, while at 60m resolution, geographical features (the river primarily, and secondarily land usage with respect to urban vs rural) dominate. The local values of Geary’s *c* support a similar trend.

Since the resolution of the Landsat sensor data is 30 *m*, we cannot readily analyze the the accuracy of our population estimation methods as a function of image resolution. We also cannot construct and evaluate complete contingency or distance maps for spatial autocorrelation analysis, because our survey data is limited to 20 of 68 sections of Bo City. Given the findings discussed above, it would useful to repeat our analysis using sensor data at different levels of resolution, using more complete survey data. For example, the interaction between spatial autocorrelation patterns for housing (i.e. structure) density, the ground-truth population density, and the estimated population density could all be examined.

### Future research

#### Simulated subsampling

One approach to studying the relationship between resolution, spatial autocorrelation, and model accuracy would be to simulate resampling of the surveyed population using a fixed grid size, perhaps with grid squares as small as 500$m^2$. The grid size must still sufficiently large to ensure that the population within each grid square is too large to be mapped onto specific dwellings that are within the square. Population maps at diverse resolutions could then be constructed by combining the populations of 2, 3, or 4 adjacent grid squares into single cells. The smaller the cell, the finer the sample granularity would be.

The Landsat measurements, which are currently averaged over the area of each section, would also have to be recalculated for each of the grid squares for each of the grid resolutions. In the bands used, the Landsat sensor (i.e. pixel) resolution of 30 *m* would still be significantly smaller than the sizes of the reduced sample grid squares. (A pixel resolution of 30 *m* is still larger than a typical residential dwelling.) The independent variable would be the number of persons per grid cell, and both global and local measures of spatial autocorrelation could be computed. This approach should disclose regions that are locally clustered and spatially correlated, as a function of grid resolution. The Landsat sensor values would also have to be recomputed, roughly matching the resolution of the resampled grid squares. Given a finer grid resolution, we could determine if the relative error *RE* for the LOOCV cross-validation decreases. It would also be possible to define training sets and test sets for conventional cross-validation testing.

Even given high-resolution subsampling, it would still not be possible to construct a complete adjacency or distance matrix for the current dataset, because only 20 of 68 sections were surveyed. But within contiguous sub-regions of Bo City, the following two questions could also be clarified: (1) Do patterns of autocorrelation in the sub-sampled ground truth population data present and/or vary as a function of resolution? (2) If so, do these patterns modify the estimated population density distributions using the Landsat data?

#### Masking section imagery

A strategy for improving model generalization would be to partially mask the imagery for each section prior to calculating the values of the covariates. The objective is to correct for the non-homogeneity of the population density within certain sections by masking (i.e. omitting) non-residential sub-areas of a section. This requires omitting pixels corresponding to areas of vegetation. This could done manually as proof of concept. Alternatively, the NDVI (normalized difference vegetation index) could be calculated for each section, and pixels that have relatively high positive values [33] could be omitted from further consideration. (Given rasters for Band 3 and Band 4, the $NDVI=(\text {Band 4} - \text {Band 3})/(\text {Band 4} + \text {Band 3})$). A limitation of this approach is that it may not mask non-residential areas that are either barren or dominated by unhealthy vegetation, but the distribution of included and excluded pixels will also be a function of the exclusion threshold selected. The index values range between − 1.0 and + 1.0. An NDVI value of zero or less means that no vegetation is present, and a maximum value of +1.0 is the strongest possible indicator of healthy vegetation at the pixel location. Here again, the objective is to demonstrate a decrease in the cross-validation error by improving compliance with the model’s assumptions.

### Alternative approaches to cross-validation

The median absolute value of the relative proportional error *RE*, as defined in 4 and enumerated in Table 9, is about 8.0%. For example, referring to Table 9 for section Roma, RE = (3818.48-3475.00)/3475.00 $\times$ 100% = 9.88%. The median absolute value of the 20 values of *RE* is 8.85%. Conversely, the sum of the estimates of the section populations in Column 6 is very close to the measured value of the total population. While some sections had a lower-than-observed population and others had a higher-than-observed population, the estimated total sum across all sections (25,856) was very close to measured population size (25,954), an error of less than 1.0%.

The generality of the model was tested using LOOCV (k-1) cross-validation. The results here were less satisfactory than for the population density ${\hat{d}}$ estimations. Although the median absolute relative error was only 11.14%, the *RE* errors of over 40% for 2 of the 20 sections and over 20% for two additional sections. A limitation of the LOOCV cross-validation paradigm was that only a single observation was available for each trial. Extending the training set would reduce the limitations imposed by the small number of 20 observations available. A larger dataset could be partitioned into multiple training sets and test sets; this would provide a far more robust approach to cross-validation.

### Alternative estimators

Finally, there is an additional consideration for which we have conducted a preliminary test. The empirical local Bayes estimator (EBL) can provide a useful and effective benchmark, but it is a controversial one [34]. As Zeugner [17] succinctly states, “It does not constitute a real prior since it involves ‘peeking’ at the data in order to formulate a prior.” Allowing for these limitations, we developed an EBL model using the data set already described. This was done using the BMS package for R [17], as was the preceding work; the spectral data subset was used, with a reduction in highly-correlated variables executed first.

**Fig. 6:**
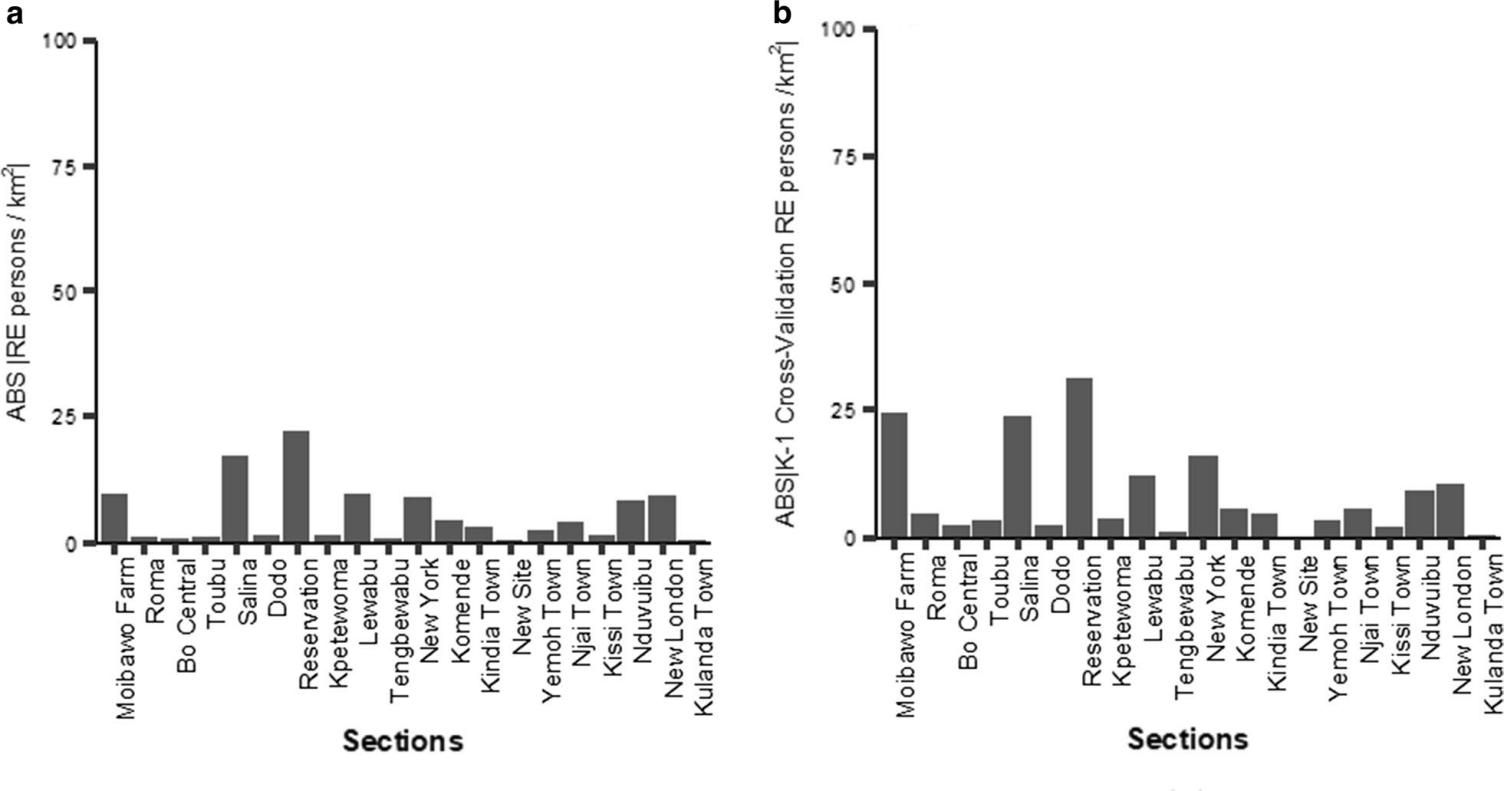
The Relative Errors *REs* for the back-transformed estimate ${\hat{d}}_i$ and cross-validation trials using a Local Empirical Bayesian estimator. The same results shown in Fig. 5a, b, except a Local Empirical Bayesian (EBL) estimator was used

**Table 11:** Measured and estimated values of population and population density using EBL estimator

y(1) Name	(2) p = Population	(3) Measured area in km$^2$	(4) d = Persons per km$^2$	(5) Estimated ${\hat{d}}$	(6) Estimated ${\hat{p}}$	(7) *RE * (%)
Moibawo Farm	135	0.5	270.04	203.5	101.75	− 24.64
Roma	139	0.04	3510.72	3353.39	134.1356	− 4.48
Bo Central	273	0.066	4137.9	4224.7	278.8302	2.10
Toubu	454	0.016	28089.66	27109.01	433.7442	− 3.49
Salina	580	0.467	1242.8	1537.59	718.0545	23.72
Dodo	597	0.049	12126.22	12417.3	608.4477	2.40
Reservation	637	2.329	273.5	188	437.852	− 31.26
Kpetewoma	640	0.197	3250.74	3374.54	664.7844	3.81
Lewabu	879	0.479	1836.16	2057.56	985.5712	12.06
Tengbewabu	1068	0.68	1571.17	1557.68	1059.222	− 0.86
New York	1088	1.513	719.3	604.88	915.1834	− 15.91
Komende	1103	0.196	5622.16	5924.81	1161.263	5.38
Kindia Town	1160	0.146	7972.46	8357.53	1220.199	4.83
New Site	1248	0.686	1818.17	1815.95	1245.742	− 0.12
Yemoh Town	1858	0.404	4602.47	4455.86	1800.167	− 3.19
Njai Town	2298	0.216	10641.33	10061.91	2173.373	− 5.44
Kissi Town	2490	0.196	12709.81	12463.32	2442.811	− 1.94
Nduvuibu	2552	0.493	5177.21	5661.29	2791.016	9.35
New London	2873	0.597	4813.01	4308.02	2571.888	− 10.49
Kulanda Town	3882	0.294	13216.15	13149.33	3865.903	− 0.51
Mean	1297.7	0.48	6180.05	6141.31	2947.829	− 0.63
Median	1078	0.35	4370.18	4266.36	1493.226	− 2.38

This table lists the (1) section name (2) measured section population (3) measured section area in $km^2$ (4) population density $d = Persons/km^2$ (5) the regression-estimated population density ${\hat{d}}$ (6) the estimated population/section $\hat{p_{i}}=Area*{\hat{d}}$. (7) the Relative Error (*RE*) for the density estimation

**Table 12:** The best regression model found by the MCMC sampler for the EBL estimator

(1) Covariates	(2) Estimated coefficient	(3) Std. error	(4) t value	(5) Pr($>|t|$)	(6) Max. Prob.	(7) VIF	(8) PIP
intercept	129.60	6.17	21.00	0.00	< .001	–	–
nb3s	1076.00	28.09	33.32	0.00	< .001	8.97	0.31
nb7v	− 1882.00	56.70	− 33.20	0.00	< .001	7.39	0.48
nb7c	61.42	4.95	12.39	0.00	< .001	1.25	0.15
ds15s	− 2112.00	109.10	− 19.36	0.00	< .001	2.17	0.22
ds35c	239.90	40.13	5.98	0.00	< .001	2.58	0.10
ch127	0.57	0.02	− 26.76	0.00	< .001	2.19	0.23
ch357	− 0.57	0.02	− 31.22	0.00	< .001	1.50	0.24

This table summarizes the best regression equation returned by the MCMC sampler for the estimation of $\sqrt{d} using the EBL estimator$. The values of the variance inflation factor (VIF) are less than 7.0, which demonstrates the low collinearity between the covariates. Four of Posterior Inclusion Probabilities (PIPs) are close to 1.0, quantifying their importance as predictive variables of $\sqrt{d}$, as discussed in the text.

In this case, a 6 variate regressor equation was found, plus the non-zero intercept. See Tables 11 and 12 for details. In Fig. 6, the EBL bar charts show both the relative errors (*RE*) for the estimations of the population density and the absolute values of the *REs* for the cross-validation tests. A comparison of Figs. 5 and 6 show that the EBL is far more effective than the conventional Bayesian model developed within. Specifically, the *RE* for the estimated population density is much lower (compare Figs. 5a, 6a). The cross-validation *RE* (Fig. 6b) is greatest for Moibawo Farm (270 persons/$km^2$) and Reservation (273 persons/$km^2$), the two sections with the lowest population densities (Table 1) and the greatest RE underestimations for cross-validation. About half of the footprint for Reservation is bright green wetlands, and Moibawo Farm is heavily forested. The cross-validation RE for Salina, which has a large industrial area surrounding the main road (the “old railroad line”), is overestimated by almost $25\%$. An interesting research question is which model will be better generalize to data sets that were *not* used to condition either model.

## Conclusions

The objective of our study was to demonstrate that it is possible to *rapidly* develop a predictive model for estimating the population density, and the contingent population count, for local neighborhoods in an urban environment using Landsat data. Although some limitations are imposed by the non-homogeneity of population density in several sections, including Reservation and Moibawo Farm, we have succeeded in this objective. An accurate 6-covariate linear multiple regression model was developed for estimating the population density *d*. Methodological improvements are also suggested, including NDVI masking of section imagery prior to variable calculation, and higher resolution subsampling of the original survey data. Although our approach will probably not be as accurate as methods using high-resolution satellite imagery, if offers a number of advantages with respect to speed and simplicity for the estimation of local populations:

1. It uses LEDAPS (Landsat Ecosystem Disturbance Adaptive Processing System) pre-processed Landsat sensor data for deriving variable values.
2. It is not necessary to manually (or automatically) extract residential structure outlines or to define GIS layers or geographical features that correlate with residential areas.
3. Only 30 m LandSat data resolution is required, not high-resolution (<10m) imagery.
4. Each of the six regression covariates selected was derived directly from Landsat sensor imagery, rather than being a composite variable, as in principal components analysis.
5. The posterior inclusion probability (PIP), calculated for each covariate, provides a measure of the variable’s information-theoretic significance within the top 1000 candidate regression models.
6. The calculations are also relatively speedy, requiring only a few minutes to run $10^6$ Markov chain Monte Carlo (MCMC) iterations and less than 30 min to execute $10^7$ iterations. All results discussed in this article are from simulations run with $10^7$ iterations, following exploratory simulations with $10^6$ iterations.

Potential strategies were discussed that will maintain the above advantages while potentially improving the accuracy and generality of the models.

## Data Availability

All data are fully available without restriction, with the relevant tabular data within the paper and its Appendices. GIS data are available on OpenStreetMap (http://osm.org/go/am_ZKeeU). Landsat imagery are available from the US Geological Survey (USGS).

## Appendices

### Appendix 1: an overview of methods for satellite-based estimations of population and urban footprint

The use of satellite imagery for appraising land utilization, including population density estimation, is not novel. A study using urban Landsat data for Sydney, Australia, demonstrated that three distinct classes of surface reflectance data corresponded to vegetation, urban residential areas, and urban non-residential areas [35]. Applying Landsat data, Forster also developed a set of pixel-based regression models for predicting the type of land cover [buildings, concrete, roads, trees, grass, water and soil] in Sydney. Correlations of up to 70% were achieved, and up to 85% for tree and road cover over extended areas [36]. More recently, Forster demonstrated that the coefficient of variation of single-band SPOT (Satellite Pour l’Observation de la Terre) HRV (High Resolution Visual) imagery could be used to estimate housing density and average house size in the area of Sydney, Australia [37]. Harvey developed regression models for estimating the population densities and total population of Australian Census Districts (CDs) from Landsat thematic mapper data [5, 38]. The first study area included 225 CDs, and the second 138 CDs, which enabled the partitioning of the data into “training” and “test” datasets. See [39] for a review of more recent “bottom-up” population estimation models using high-resolution satellite imagery.

Satellite imagery has also been used to develop models to estimate the spatial footprint of cities in low- and/or middle-income countries, and their evolution over time. Wilson and his collaborators have developed a series of temporal models for Sierra Leone demonstrating how Land Use and Land Cover (LU/LC) evolve as a function of civil conflict. The spatial LU/LC models developed for the capital city of Freetown, and the metropolitan cities of Bo and Kenema, encompass three multi-year intervals preceding, during, and after a period of civil strife [27]. In a study of the Indonesian island of Java, Patel et al [40] established that Landsat imagery is valuable for tracking urbanization—that is, the growing footprint of cities, rather than the rise in the number of inhabitants—in growing cities. In this study, the Landsat database was accessed via the Google Earth Engine (GEE). Trianni [41] also used GEE to generate estimates of human population size using Landsat data in Brazil, China, and Indonesia. Son [42] and his collaborators used Landsat data to track the expanding footprint of the capital city Tegucigalpa; while Linard et al. [43] tracked changes in the human population over a ten-year period on the Kenyan coast, integrating data from multiple sources, including Landsat imagery and historical census data.

#### GUF

Aerial imagery, including hyperspectral datasets, can now be supplemented by high-resolution satellite measurements using SAR (*Synthetic Aperture Radar*) imaging [44]. The highly automated GUF (*Global Urban Footprint*) utilizes SAR amplitude data from the TerraSAR-X and TanDEM-X satellites, in combination with texture maps derived from the SAR amplitude data [31]. Strong primary and secondary backscattering from vertical structures result in characteristic bright spots in the texture maps. Conversely, natural phenomena such as tall trees and rock formations can result in false positives, while dense groups of low flat-roof houses may produce false negatives. An unsupervised classification system, based on *Support Vector Data Description* (SVDD), used these paired distributions to identify built-up regions characterized by vertical buildings, with a spatial resolution of 12 m.

#### GHSL

The GHSL (Global Human Settlement Layer) [45] was derived by processing archived Landsat data. Data collected by the MS (*Multispectral Scanner*), TM (*thematic mapper*), and ETM (*Enhanced thematic mapper*). (In our current study, only TM data was used.) The resulting sequence of maps shows dramatic changes in patterns of urban growth over a 40-year period.

#### HRSL

The HRSL (*High Resolution Settlement Layer*) [29, 46] effort is a fourth project for mapping the urban footprint. In this effort, man-made buildings were individually identified from high-resolution satellite imagery. Census data was then proportionally mapped onto the urban areas of interest. This proportional allocation was validated by comparison with census data for Ghana, Malawi, and Vietnam. In addition, the predicted spatial incidence of Malawi household residences was verified using household survey data, although no attempt was made to estimate the number of occupants in specific residences.

#### A novel approach to estimating the urban footprint

Deville [47] and his collaborators analyzed two large datasets of mobile phone (MP) calls made in France and Portugal during 2007 and 2008. Using the MP data, MP usage was estimated as a function of time of day, season, and location. Usage was scaled by the census-estimated national population to estimate the local population densities. The estimated population densities compared favorably to estimates derived independently from multiple sources.

#### Accuracy and image resolution

In a comprehensive review of *low resolution* (LR) and *high resolution* (HR) mapping techniques, [31] found broad agreement between the corresponding maps. However, HR methods including GUF and GHSL were superior with respect to “completeness and precision,” including more accurate representation of the boundaries of large urban areas where the density of residential structures are decreasing. In rural sub-Sahara Africa, these HR methods were the only systems that successfully classified at least some of the small, disconnected settlements in rural areas.

#### The challenge of improvised settlements

Unplanned improvised settlements [3], temporary refugee camps [1] and other informal communities pose special challenges to almost any population mapping method. First, the clusters of improvised structures may be difficult to delineate and extract from aerial imagery, although our own methodology does not utilize feature extraction. Second, the presumed relationship between rooftop area and occupancy levels may be violated; this can make proportional allocation of the population more difficult, a method used in several of the preceding studies [29, 47]. As an example of the latter, a specific residence in Bo was included that had a measured rooftop area of 8 meters on a side, or about 25 feet $\times$ 25 feet. Seven families, for a total of 62 inhabitants, used this residence as sleeping quarters. This serves as an example of extreme resource-poverty, where a single family may be forced to live in one room, with multiple small rooms within a single residence [Rashid Ansumana, personal communication.]

### Appendix 2: variable definitions

This Appendix contains the definitions for all of the candidate covariates used in this paper. See Table 5. There are a number of differences between the dataset in Table 6 and the list of candidate covariates summarized in [5]. For the normalized variable $nb_i$, we used a min-max normalization, rather than a z-score transform. Based on preliminary simulations, the min-max transform is equally or more effective as a covariate than a z-score transforms. (The latter was calculated as the mean z-score of the pixels in a section, where the individual pixel z-scores were calculated relative to the grand mean and $\sigma$ for all sections combined.) A second difference was the introduction of a modified notation to explicitly differentiate between the non-spectral ratios of means $r.sp_{ij}$ and the spectral ratio operator $r_{ij}$.

#### Non-spectral variables

The non-spectral variables consist of the means, standard deviations, and variances of $b_{i}$ within a single section, and the specified ratios of these values [Table 3].

The mean band values $b_i$ and textures:

5 $$\begin{aligned} \mathbf {b}_{\mathbf {i}}&={\frac{\mathbf {1}}{\mathbf {p}}{\sum _{\mathbf {n=1}}^{\mathbf {p}}\mathbf {b}_{\mathbf {i}_\mathbf {n}}}} \end{aligned}$$

6 $$\begin{aligned} \mathbf {bs}_{\mathbf {i}}&={\sigma _{\mathbf {b}_{\mathbf {i}}}} \end{aligned}$$

7 $$\begin{aligned} \mathbf {bv}_{\mathbf {i}}&=\mathbf {var}(\mathbf {b}_{\mathbf {i}}) \end{aligned}$$

8 $$\begin{aligned} \mathbf {bc}_{\mathbf {i}}&= {\frac{\sigma _{\mathbf {b}_{\mathbf {i}}}}{\mathbf {b}_{\mathbf {i}}}}\nonumber \\&= \frac{{\mathbf {bs}}_{\mathbf {i}}}{{\mathbf {b}}_{\mathbf {i}}} \end{aligned}$$

The square of $b_{ij}$:

9 $$\begin{aligned} \mathbf {s}_{\mathbf {i}}&= {\mathbf {b}_{\mathbf {i}}^2} \end{aligned}$$

The non-spectral cross-product of $b_i$ and $b_j$:

10 $$\begin{aligned} \mathbf {p}_{\mathbf {ij}}&= {\mathbf {b}_{\mathbf {i}}\times {\mathbf {b}_{\mathbf {j}}}} \end{aligned}$$

The non-spectral ratios of $b_i$ and $b_j$:

11 $$\begin{aligned} \mathbf {r.re}_{\mathbf {ij}}&= {\frac{\mathbf {b}_{\mathbf {i}}}{\mathbf {b}_{\mathbf {j}}}} \end{aligned}$$

The difference/sum of $b_i$ and $b_j$:

12 $$\begin{aligned} \mathbf {d}_{\mathbf {ij}}&= {\frac{\mathbf {b}_{\mathbf {i}}-\mathbf {b}_{\mathbf {j}}}{\mathbf {b}_{\mathbf {i}}+\mathbf {b}_{\mathbf {j}}}} \end{aligned}$$

#### Spectral transforms

The non-spectral transforms are the mean values $b_i$ and associated measures of variation [standard deviation, variance, and coefficient of variation] of the elementary pixel measurements ($b_i$); as well as the squares $s_i$, products $p_{ij}$, ratios $r.re_{ij}$ and sums/differences $d_{ij}$ of the mean values $b_i$.

The spectral transforms, which we will define next, are pixel-level functions. Unlike the non-spectral transforms, the spectral transforms are functions of the ratios, products, or differences of individual pixels, rather than functions of the mean values of band measurements.

Let *p* denote the number of pixels in section *i*, and let *ij* denote a specific pixel *j* in band *i*. Let $Max_i$ be the maximum value of $b_{ij}$ in section *i*, and $Min_i$ be the minimum value of $b_{ij}$. $nb_{i}$ is defined as the mean value of the min-max normalized values of section *i*. Specifically:

13 $$\begin{aligned} \mathbf {nb}_{\mathbf {i}}&= {\frac{1}{\mathbf {p}_\mathbf {i}}{\sum _{\mathbf {j=1}}^{\mathbf {p}} \frac{\mathbf {Max}_{\mathbf {i}}-\mathbf {b}_{\mathbf {ij}}}{\mathbf {Max}_{\mathbf {i}}-\mathbf {Min}_{\mathbf {i}}}}} \end{aligned}$$

From the preceding equation, each of the *p* normalized values in the summation term is a number between 0 and 1. The range of $nb_{i}$, which is the mean of these values, will necessarily fall between 0 and 1. The standard deviation $nbs_i$, variance $nbv_i$, and CV $nbc_i$ of $nb_i$ are defined as:

14 $$\begin{aligned} \mathbf {nbs}_{\mathbf {i}}&= {\mathbf {sd}(\mathbf {nb}_{\mathbf {i}})}\nonumber \\&= {\sigma _{\mathbf {b}_{\mathbf {i}}}} \mathbf {nbv}_{\mathbf {i}} \nonumber \\&= {\mathbf {var}(\mathbf {nb}_{\mathbf {i}})} \end{aligned}$$

15 $$\begin{aligned} {\mathbf {nbc}_{\mathbf {i}}}&= {\frac{\sigma _{\mathbf {nb}_{\mathbf {i}}}}{\mathbf {nb}_{\mathbf {i}}}} \nonumber \\&= {\frac{\mathbf {nbs}_{\mathbf {i}}}{\mathbf {nb}_{\mathbf {i}}}} \end{aligned}$$

For a given section, the spectral function $r_{ij}$ is the mean ratio of the paired pixel values in bands *i* and *j*. Each is assumed to have *p* pixels (TM measurements). The variable $bpx_{i_n}$ is the value of pixel *n* in band *i*, and $bpx_{j_n}$ is the value of pixel *n* in band *j*.

16 $$\begin{aligned} \mathbf {r}_{\mathbf {ij}}&= {\frac{\mathbf {1}}{\mathbf {p}}{\sum _{\mathbf {n=1}}^{\mathbf {p}} \frac{\mathbf {bpx}_{\mathbf {i}_\mathbf {n}}}{\mathbf {bpx}_{\mathbf {j}_\mathbf {n}}}}} \end{aligned}$$

The standard deviation of $r_{ij}$ is denoted by $rs_{ij}$, the variance by $rv_{ij}$, and the coefficient of variation by $rc_{ij}$. The variable $ds_{ij}$, the mean of the paired ratios of the differences and sums of the pixel magnitudes in bands *i* and *j*, is computed similarly to $rs_{ij}$.

17 $$\begin{aligned} \mathbf {ds}_{\mathbf {ij}}&= {\frac{\mathbf {1}}{\mathbf {p}}{\sum _{\mathbf {n=1}}^{\mathbf {p}} \frac{\mathbf {bpx}_{\mathbf {i}_\mathbf {n}}-\mathbf {bpx}_{\mathbf {j}_\mathbf {n}}}{\mathbf {bpx}_{\mathbf {i}_\mathbf {n}}+\mathbf {bpx}_{\mathbf {j}_\mathbf {n}}}}} \end{aligned}$$

#### Cylindrical coordinate transforms

The cylindrical coordinate transforms are denoted by $ch_{ijk}$ and the rectangular coordinate transforms by $rh_{ijk}$. In either case, 3 of the 7 available bands were assigned to the red (*i*), green (*j*), and blue (*k*) channels. The 3 normalized mean band values are then represented as the coordinates in a Cartesian cube. Via projection onto the chromatic plane, each color triplet is transformed into a single hexagonal number; this scalar value is the candidate covariate for the cylindrical coordinate transform.

The transform $ch_{ijk}$ is calculated using Eqs. (12) and (13) in Hanbury [2008]. The rectangular coordinate transforms $rh_{ijk}$ is calculated using the piecewise hue definition in Eq. (4), reference [Hanbury and Serra, 2003]. Prior to either transform, the data is normalized so that the maximum value of the data maps onto the red channel *i* was 255.

### Appendix 3: Bayesian mixture analysis

The *Bayesian Model Selection* (BMS) R library (package) was used to develop and run the regression models [17–19]. BMS implements a Bayesian Model Averaging (BMA), based on the premise that a properly weighted mixture of Bayesian models can estimate a desired set of observations more accurately than a single regression model can. The number of best models for which the model parameters are saved is user-defined, and were conservatively set to 1000 in our simulations, rather than to the default value of 200. The sampler can be set to retain data for the best regression model only, but it would no longer be possible to graphically verify the convergence of the posterior model probabilities. or to obtain predictions based on an average mixture of models.

Define a regression model $M_j$ for estimating the dependent variable *y*, $\beta _{0}$ is the constant intercept term, and $x_1$, $x_2$, ...$x_k$ are the *k* covariates. See [19], Eq (1).

18 $$\begin{aligned} \begin{aligned} {\mathbf {y}}&= \beta _{0}+\beta _{1}x_{1}+\beta _{2}x_{2}+\cdots +\beta _{k}x_{k}+\epsilon \end{aligned} \end{aligned}$$

Let $\beta$ be the vector of parameters (regression coefficients) from model $M_j$, and $Pr(M_j)$ be the probability that $M_j$ is the true model. The estimated posterior means of ${\widehat{\beta }}=(\widehat{\beta _0},\widehat{\beta _1},...,\widehat{\beta _k})$ are:

19 $$\begin{aligned} \begin{aligned} E[{\widehat{\beta }}|D]=\sum _{j=1}^{2^k}{{\widehat{\beta }}Pr(M_{j}|D)} \end{aligned} \end{aligned}$$

Given *k* covariates in each candidate regression model $M_j$, there will be $2^k$ models in the model space. For values of *k* exceeding 14, an exhaustive enumeration of candidate regression models is computationally challenging, and a heuristic algorithm is usually required. Our simulations were run using the birth-death MCMC sampler, and the random number generator seed was explicitly specified in order to permit replicability. The sampler continually draws random models, and moves to the latest model if its marginal likelihood exceeds that of the current model. Let *X* be a specific subset of *k* covariates, *y* the fixed set of *N* observations. The number of times each candidate regression model is retained converges to the distribution of the *Posterior Model Probability* $p(M_{i}|y,X)$ [19].

At the conclusion of the stochastic simulation, the single best model can be identified and fitted using the point estimates of the specified coefficients as a linear multivariate regression model. This simplifies the use of scale transforms for the dependent variable (log, inverse, square root) and their associated back-transforms; the back-transformed regression model can then be cross-validated by using the k-1 “Leave One Out” Cross-Validation (LOOCV) [21] paradigm that will be discussed subsequently.

#### Selection of $\beta$ priors

As will any Bayesian regression model, the choice of the priors for the $\beta$ vector is critical, because the choice of priors determine model selection and shrinkage. In the BMS software, the Bayesian normal-conjugate linear model defines the multiple regression model, and “Zellner’s g-prior” defines the priors for the $\beta$s (i.e. the regression coefficients) [19, 48]. We set the hyperparameter *g*^1^ equal to the *Unit Information Prior* (UIP), the default value, which proved to be effective. Because the number of candidate regressors greatly exceeded the maximum sample size of 20, the prior for the model size was set equal to $k=20/2=10$. See [34] for a discussion of the rationale for the UIP prior and others g-priors, and [17] pp. 16–18 for additional discussion on the choice of priors.

#### Determination of best single model

At the conclusion of the stochastic simulation, the single best model (the “top model” [17]) can also be exported as a linear model estimated under Zelner’s g-prior, and used for subsequent analysis. This model will yield a limited number of significant regression coefficients that can be analyzed further using conventional OLS analysis. The significance of the individual covariates is quantified by the PIP, which is equivalent to the Bayesian information criterion (BIC) developed by Schwarz [26]. A dynamic list of the best 1000 regression models found by the MCMC sampler was maintained, and this permitted the analysis of convergence during the iterative process. The number of models tracked is user-specified.

## Abbreviations

BIC: Bayesian information criterion
CV: Coefficient of variation
DOF: Degrees of freedom
EBL: Empirical local Bayes estimator
GIS: Geographic information system
LEDAPS: Landsat Ecosystem Disturbance Adaptive Processing System
LISA: Local Indicators of Spatial Association
LOOCV: Leave one out cross-validation
LU/LC: Land-use/land-cover
MCMC: Markov chain Monte Carlo
NDVI: Normalized Difference Vegetation Index
NIR: Near infrared
PCA: Principal components analysis
PIP: Posterior inclusion probability
R, G, B: Red, green, blue
SLC: Scan line corrector
TM: Thematic mapper
TOA: Top of the atmosphere
USGS: U.S. Geological Survey
VIF: Variance inflation factor
%RE: Relative proportional error
